# Supplementary material for: A Global Screen for Assembly State Changes of the Mitotic Proteome by SEC-SWATH-MS
Source: Cell Syst. 2020 Feb 26;10(2):133–155.e6. doi: 10.1016/j.cels.2020.01.001 (PMC7042714; doi:10.1016/j.cels.2020.01.001)

Q9UBN7 | HDAC6_HUMAN | HDAC6 KIAA0901 JM21
Monomer MW [kDa]: 131.419 Monomer expected elution fraction: 38

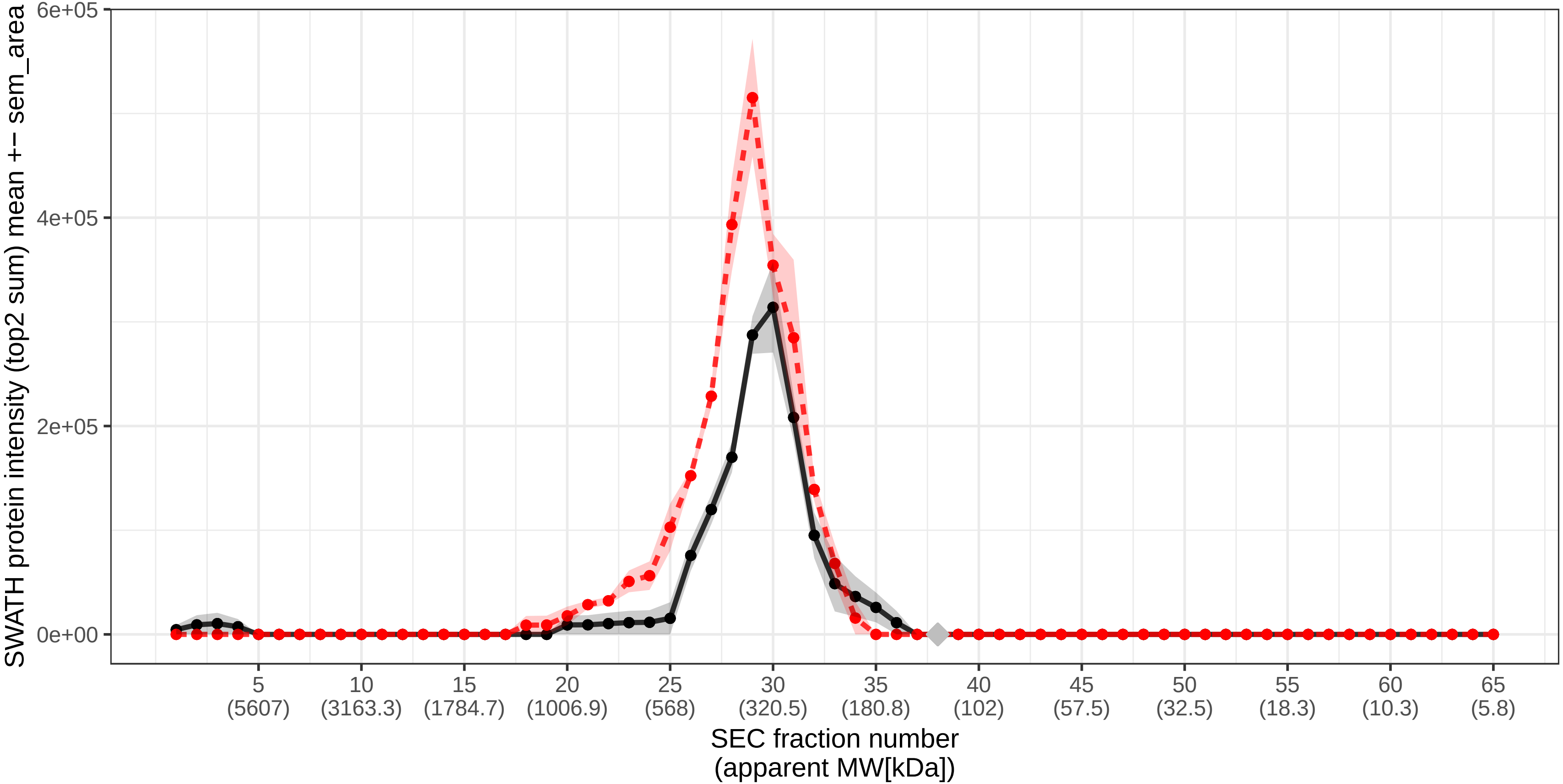

Supplement: Data S1. SEC-SWATH-MS Protein Chromatograms, Related to Figure 1 [file mmc6.zip › SECchrom_Q9UBN7_HDAC6_HUMAN_HDAC6_KIAA0901_JM21.pdf]
